# Spinal nomenclature and traffic lights – A need to standardize

**DOI:** 10.1016/j.inpm.2022.100084

**Published:** 2022-03-17

**Authors:** Alexander Shustorovich, Zirong Zhao, Mark Wallace

**Affiliations:** a Division of Pain Medicine, Department of Anesthesiology, University of California, San Diego, USA; b Department of Neurology, VA Healthcare Center, District of Columbia, Washington, USA

A nomenclature for spinal imaging and interventional procedural reporting is long overdue as standardization in medicine dictates both better outcome and safe treatment [1]. Over the last two decades there has been an eruption of various treatment modalities for chronic spine pain practiced by interventionalists from many specialties with diverse training background. This has introduced heterogeneity in the terminology used to describe techniques. Although the advent of new technologies and procedures provide patients with more personalized care, the lack of standardized nomenclature introduces gaps in information exchange among practitioners leading to increased procedure risks and reduced efficacy.

As eloquently stated by Gill et al., “the need for standardized terminology for interventional spine care is manifest”. Classification systems are commonly found throughout medical and surgical specialties, such as the Mallampati scores for airway assessment in anesthesia, FIM scores for functional independence assessment in rehab medicine, fracture classifications in orthopedics, spinal imaging pathology classifications in interventional spine and radiology, and the New York Heart Association (NYHA) classification system for heart failure [[2], [3], [4], [5], [6], [7]]. This is not an exhaustive list of validated classification systems, but this list serves as a reminder that these systems ubiquitously provide objective means for predicting risk, guiding treatment, and prognosticating outcomes. Yet, such systems in interventional pain medicine are scant and poorly adopted.

Current interventional procedural reporting is subject to heterogenous terminology. “Terms such as off-midline, paramedian, paracentral, parasagittal, gutter, and paraforaminal” are ambiguous and leave too much room for individual interpretation [8]. To date there are no well-established nomenclatures systems for spinal interventions and ultimately providers rely on anatomical descriptions from societal consensus guidelines [9,10]. However, anatomical localization does not always account for inter-patient anatomical variations.

The “stop-light” system proposed by this nomenclature is a great visual representation of “safe”, “caution”, and “danger” zones for needle placement. Universal adoption of this system has the potential to improve not only within-provider outcomes, but also inter-provider outcomes by establishing a patient-specific “road map” for successful injection therapy. Following suite, current and future trainees may learn techniques with greater validity and reliability. For example, localization of needle tips for cervical transforaminal epidural access will avoid positioning needles in areas of known vascular contrast uptake and avoid potential vascular injury. Inherently, an established nomenclature system can reduce procedure risk, procedure time, X-ray exposure, and maintain positive patient outcomes for repeat interventions.

The classification of thoracic spinal procedures, such as medial branch neurotomy, is particularly difficult due to significant anatomical variation. Targeting of the posterior sacroiliac articulating lateral branches is similarly troublesome. We understand the decision to avoid proposing a nomenclature for these regions of the body, however, there may be a missed opportunity in doing so. This can be regarded as a pitfall to the proposed system; where a standardized nomenclature intended for improved reliability and validity is invalidated by the intrinsic variability of the targets. Yet, it may still be worth pursuing in order to enhance within-patient and inter-provider outcomes. By establishing consistent thoracic and sacroiliac targets for individual patients we can effectively mitigate the issue of variability for the medial and lateral branches, respectively. Providers will then be able to repeat neurotomies with known accuracy and predictable outcomes. Additionally, an established nomenclature system will facilitate future prospective research and a standardized database for interventional spine procedures can be created. Analysis of this database can provide big data for safety, efficacy, and outcomes, which could translate to improved insurance reimbursement (e.g., thoracic and sacroiliac neurotomy).

Likely the major limitation and hardship will be the universal adoption of this nomenclature system. The benefit is clear. We commend the insight of the authors in developing a system as simplified as possible. Implementation may take several generations of interventional pain providers, but this proposal is the first step in the right direction. It may be a long road, but at least now we have a map.

## References

[1] Rozich J.D., Howard R.J., Justeson J.M., Macken P.D., Lindsay M.E., Resar R.K. (2004 Jan). Standardization as a mechanism to improve safety in health care. Joint Comm J Qual Saf.

[2] Lee Anna, Fan Lawrence T.Y., Gin Tony, Karmakar Manoj K., Ngan Kee, Warwick D. (2006). A systematic review (Meta-Analysis) of the accuracy of the Mallampati tests to predict the difficult airway. Anesth Analg.

[3] Chumney D., Nollinger K., Shesko K., Skop K., Spencer M., Newton R.A. (2010). Ability of Functional Independence Measure to accurately predict functional outcome of stroke-specific population: systematic review. J Rehabil Res Dev.

[4] Egol K.A., Koval K.J., Zuckerman J.D., Ovid Technologies, Inc (2015).

[5] Pfirrmann C.W., Metzdorf A., Zanetti M., Hodler J., Boos N. (2001 Sep 1). Magnetic resonance classification of lumbar intervertebral disc degeneration. Spine.

[6] Modic M.T., Steinberg P.M., Ross J.S., Masaryk T.J., Carter J.R. (1988). Degenerative disk disease: assessment of changes in vertebral body marrow with MR imaging. Radiology.

[7] Bjork J.B., Alton K.K., Georgiopoulou V.V., Butler J., Kalogeropoulos A.P. (2016 Jul). Defining advanced heart failure: a systematic review of criteria used in clinical trials. J Card Fail.

[8] Gill J.S., Stojanovic M., Simopoulos T.T. (2016 May). In defense of the meticulous: a case against ambiguity, and a time to standardize. Pain Physician.

[9] Hurley R.W., Adams M.C.B., Barad M. (2022). Consensus practice guidelines on interventions for cervical spine (facet) joint pain from a multispecialty international working group. Reg Anesth Pain Med.

[10] Cohen S.P., Bhaskar A., Bhatia A., Buvanendran A., Deer T., Garg S., Hooten W.M., Hurley R.W., Kennedy D.J., McLean B.C., Moon J.Y., Narouze S., Pangarkar S., Provenzano D.A., Rauck R., Sitzman B.T., Smuck M., van Zundert J., Vorenkamp K., Wallace M.S., Zhao Z. (2020 Jun). Consensus practice guidelines on interventions for lumbar facet joint pain from a multispecialty, international working group. Reg Anesth Pain Med.

